# Elevated atmospheric CO_2_ concentration triggers redistribution of nitrogen to promote tillering in rice

**DOI:** 10.1002/pei3.10046

**Published:** 2021-05-08

**Authors:** Juan Zhou, Yingbo Gao, Junpeng Wang, Chang Liu, Zi Wang, Minjia Lv, Xiaoxiang Zhang, Yong Zhou, Guichun Dong, Yulong Wang, Jianye Huang, Dafeng Hui, Zefeng Yang, Youli Yao

**Affiliations:** ^1^ Jiangsu Key Laboratory of Crop Genetics and Physiology/Co‐Innovation Center for Modern Production Technology of Grain Crops College of Agriculture Yangzhou University Yangzhou China; ^2^ Key Laboratory of Plant Functional Genomics of the Ministry of Education/Jiangsu Key Laboratory of Crop Genomics and Molecular Breeding College of Agriculture Yangzhou University Yangzhou China; ^3^ Lixiahe Agricultural Research Institute of Jiangsu Province Yangzhou China; ^4^ Department of Biological Sciences Tennessee State University Nashville Tennessee USA

**Keywords:** atmospheric CO_2_, distribution, gene expression, nitrogen, rice, tiller

## Abstract

Elevated atmospheric CO_2_ concentration (eCO_2_) often reduces nitrogen (N) content in rice plants and stimulates tillering. However, there is a general consensus that reduced N would constrain rice tillering. To resolve this contradiction, we investigated N distribution and transcriptomic changes in different rice plant organs after subjecting them to eCO_2_ and different N application rates. Our results showed that eCO_2_ significantly promoted rice tillers (by 0.6, 1.1, 1.7, and 2.1 tillers/plant at 0, 75, 150, and 225 kg N ha^−1^ N application rates, respectively) and more tillers were produced under higher N application rates, confirming that N availability constrained tillering in the early stages of growth. Although N content declined in the leaves (−11.0 to −20.7 mg g^−1^) and sheaths (−9.8 to −28.8 mg g^−1^) of rice plants exposed to eCO_2_, the N content of newly emerged tillers on plants exposed to eCO_2_ equaled or exceeded the N content of tillers produced under ambient CO_2_ conditions. Apparently, the redistribution of N within the plant *per se* was a critical adaptation strategy to the eCO_2_ condition. Transcriptomic analysis revealed that eCO_2_ induced less extensive alteration of gene expression than did N application. Most importantly, the expression levels of multiple N‐related transporters and receptors such as nitrate transporter NRT2.3a/b and NRT1.1a/b were differentially regulated in leaf and shoot apical meristem, suggesting that multiple genes were involved in sensing the N signal and transporting N metabolites to adapt to eCO_2_. The redistribution of N in different organs could be a universal adaptation strategy of terrestrial plants to eCO_2_.

## Graphical abstract

Elevated atmospheric CO_2_ significantly lowers N content in leaf and sheath of rice plants, but raises the N content in the newly emerged tillers. Orchestrated with differential expression of multiple glutamate receptors, ammonium transporters, nitrate transporters, and peptide transporters between leaf and shoot apical meristem, the meristem zone maintains high N content to promote tillering under higher CO_2_ conditions.

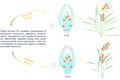

## 1 INTRODUCTION

The rise of atmospheric carbon dioxide (CO_2_) concentration has been a serious global climatic issue for decades. Despite the adoption of the Kyoto Protocol in 1997, atmospheric CO_2_ concentration ([CO_2_]) shows no sign of slowing down and reached a climax of 417.69 ppm in March 2021 (ESRL, Mauna Loa, Hawaii, USA). The increase in CO_2_ and other greenhouse gasses has increased the global mean temperature and the frequency of extreme weather events. These changes will increase crop disease and pest occurrences and reduce crop productivity, quality, and stability (Easterling & Apps, 2005; Li et al., 2015; Rosenzweig et al., 2014; Wang, Hasegawa, et al., 2019). It seems that only the rice and soybean crops may marginally benefit from elevated atmospheric CO_2_ (eCO_2_) (Ainsworth, 2008; Kimball, 2016; Long et al., 2006; Usui et al., 2016; Zhao et al., 2017). To mitigate the climate change threat and secure crop production, it is critically important to breed new crop varieties adapted to the future [CO_2_] and temperature conditions, and to develop a novel cultivation management system to take advantage of the CO_2_ fertilization effect (Long et al., 2004). However, genes responsive for eCO_2_ adaptation have not been extensively explored for breeding purposes (Hasegawa et al., 2019; Morita et al., 2015; Nakano et al., 2017). Top priority should be given to those yield‐limiting agronomic traits that showing a beneficial response to eCO_2_.

Rice (*Oryza sativa* L.) is a staple cereal crop for more than half of the world population, especially in the densely populated Asian regions. Despite various responses of productivity to eCO_2_ by different crops, it is generally concluded that rice marginally benefits from CO_2_ fertilization in temperate and tropical regimes (Kimball, 2016; Rosenzweig et al., 2014; Ruiz‐Vera et al., 2013). Previous studies have reported that the eCO_2_ increases the grain yield of rice (Hasegawa et al., 2017). For example, a multiple‐year free‐air CO_2_ enrichment (FACE) field experiment conducted at different locations with various *indica*, *japonica*, and hybrid rice varieties found that eCO_2_ at 550 ppm enhances rice grain yield by 5~35% compared to ambient CO_2_ levels (Kim, Lieffering, Kobayashi, Okada, Mitchell, et al., 2003; Kimball, 2016). Among the four major components of rice yield (panicles per area, spikelets per panicle, seed setting rate (%), and grain weight), eCO_2_ consistently increases panicles per area, while the other three yield components show both negative and positive responses (Huang et al., 2004; Hasegawa et al., 2019; Kim et al., 2003; Lai et al., 2014). The panicles per area is a fundamental factor determined at the earlier stages of rice growth. It impacts the other three yield components at later stages of growth and significantly influences final grain yield. Therefore, it is always a top priority to achieve an optimal panicle number in rice production management.

Rice plants produce tillers (branches) in addition to the main stem that may eventually develop into panicle florescences for grain yield (Wang & Li, 2011). The tiller number of a rice plant is not only a basis for panicle number, but also an indicator of plant growth status. Rice plants with more tillers at the early stage usually indicate they are on a healthy developmental path toward higher yield. Tiller number is also sensitive to CO_2_ change and shows a significant response to eCO_2_ (Huang et al., 2004; Kim et al., 2003). Since the discovery of the MONOCULM 1 (MOC1) gene in tillering regulation (Li et al., 2003), recent advances in molecular genetics have revealed that more than 60 genes are involved in the regulation of tiller production in rice plants (Wang et al., 2018; Wang & Li, 2011). However, most of the knowledge is derived from mutant or gene manipulation experiments and there is limited information available on how these genes coordinate in a regular variety (Zhang et al., 2019).

Nitrogen (N) is a major macronutrient that constrains tiller growth. Previous studies found that eCO_2_ alters the element stoichiometry in many plants. Especially consistent was a reduction in N content in plants as diverse as grasses, crops, and trees (Deng et al., 2015; Luo et al., 2006; Norby et al., 2010). Insufficient N availability is a constraint on the growth of perennial grasses in response to eCO_2_ (Mueller et al., 2013; Reich et al., 2006). Crops and model plants also display interactions between their N requirements and eCO_2_, though the details of the interactions differ (Andrews et al., 2019; Bloom, 2015; Rubio‐Asensio & Bloom, 2017; Stitt & Krapp, 1999; Wang et al., 2010).

Elevated atmospheric CO_2_ reduces the N content in rice plants (Kim et al., 2003; Lieffering et al., 2004; Makino et al., 1997; Wang, Liu, et al., 2019; Zhang et al., 2013). Low N content in rice plants is reported to inhibit tiller occurrence (Jiang et al., 1997). Despite the expected inhibition of tillering due to reduced N content in rice plants under the eCO_2_, multiple studies have reported that tillers are promoted by eCO_2_ (Huang et al., 2004; Jiang et al., 2020; Jitla et al., 1997; Yang et al., 2007). However, the underlying physiological and molecular mechanisms remain unclear in those studies.

In this study, we investigated the interactive effects of CO_2_ level and N application rate on rice tillering using growth chambers. We hypothesized that eCO_2_ would reduce plant N content, but N distribution in plants would change in favor of tillering under the eCO_2_ condition. Our objectives were 1) to analyze the interaction effect of eCO_2_ and N application rate on rice tillering at the early stage; 2) to detect changes in the N distribution among different plant organs; and 3) to investigate changes in gene expression in response to eCO_2_ at different levels of N availability. Understanding the molecular adaptation mechanisms of rice to eCO_2_ would directly enable breeders to target certain genes to generate new varieties and develop new approaches to mitigate the effect of eCO_2_. The mechanism derived here may also help interpret the adaptation of other terrestrial plants to eCO_2_.

## 2 MATERIALS AND METHODS

### 2.1 Rice material and culture conditions

The experiment was conducted in two growth chambers (CMP6050, Conviron) at the Yangzhou University in Yangzhou, Jiangsu Province, China. Japanese rice variety Nipponbare (*Oryza sativa*, ssp. *japonica*) was used in the experiments. Nipponbare is widely used in rice production, breeding, and molecular genetics studies. It was the first rice variety genome to be sequenced deeply and serves as a monocot model plant (Matsumoto et al., 2016; Sasaki, 2005).

Rice seeds were acquired from Yangzhou Agricultural Research Center and surface sterilized with the disinfectant prochloraz (Jinbao, Yangzhou Suling Agricultural Chemicals, Jiangdu, China) following the supplier's recommendations. Seeds were immersed in tap water for 48 h before germination at 32°C and 85% humidity in an incubator for 15 h, then at 28°C until shoots and roots reached 0.5~1 cm. The germinated seeds were screened for uniformity and planted in plastic trays of soil at a spacing of 3.5 cm × 3.2 cm, with each tray containing 96 plants. The tray was filled with premixed soil, 12 cm in depth (Zhang et al., 2019), and prepared in a similar way as done in field nurseries for seedlings.

During the growth period, the soil was checked three times a day to keep its moisture at saturation but without a persistent water layer. The temperature of the growth chamber was preset to 30/22°C (actual records varied in a ±0.67°C range) in a 12/12 h day/night regime. The illumination was set at 800 μmol photons m^−2^ s^−1^ (actual records varied by ±58 μmol photons m^−2^ s^−1^) during the day and 0 at night. The relative humidity was set at 70% (actual records varied between 65–80%).

### 2.2 Experimental design

This experiment used a split‐plot experimental design with four replications. The main treatment factor was [CO_2_] and subplot treatment factor was N application rate. There were two [CO_2_] levels: ambient CO_2_ (400 μmol mol^−1^) and eCO_2_ (600 μmol mol^−1^). Since we have two independent growth chambers, we set up one chamber for ambient CO_2_ and the other for eCO_2_ treatments, and repeated the whole experiment in each chamber four times. Each repeat was considered as one replication. The eCO_2_ treatment was achieved by pumping in industrial‐grade pure CO_2_ to a partial pressure of 600 μmol mol^−1^ (actual record range of 595–640 μmol mol^−1^ with a mean of 608.9 μmol mol^−1^), and the ambient condition had a CO_2_ partial pressure range of 405 to 430 μmol mol^−1^, with a mean of 410.6 μmol mol^−1^ during the growth period. As we focused on the adaptation of rice plants to eCO_2_ at the early seedling stage, we initiated eCO_2_ treatment from seeding. N application rates included four levels: 0 (N0), 5 (N5), 10 (N10), and 15 (N15) kg N 666.7 m^−2^, which were equivalent to 0, 75, 150, and 225 kg N ha^−1^, respectively, comparable to the general range of N fertilization rates used in the region at this stage of growth. Half of the N fertilizer (urea, 46% N) was premixed into the soil three days prior to seeding, and the remaining portion was top‐dressed in a water‐solution at true leaf‐ages of 1, 2, 3, and 4. Each addition was at a rate of 12.5% of the total N application. Together, there were eight treatments, considering two levels of [CO_2_] and four levels of N application rate within each level of [CO_2_], and each treatment‐level combination included two plastic trays comprising 192 plants.

### 2.3 Measurements of total N and carbohydrates content

Leaf‐age, plant height, and tiller numbers were measured at true leaf‐age four and six. Tiller occurrence percentage was calculated as the total tiller number divided by the main stem number per tray, reflecting tillering consistency among the plants at the 4th true leaf emerging stage as the plant starts to tiller. Plants were harvested at true leaf‐age six for biomass measurement (at 25–27 days after seeding). The shoot parts of plants were separated into leaf, sheath, and newly emerged tillers (those with less than three true leaves) for N content analysis using the Kjeldahl method with an automatic Kjeltec 8400 Analyzer Unit (Foss Analytical AB) following the manufacturer's recommendations (Zhang et al., 2019). For carbohydrate measurement, plants were further separated into newly emerged tillers, leaves (leaf positions 1–5), or sheaths (upper and lower halves). Carbohydrates were measured using the anthrone reagent method (Zhang et al., 2019).

### 2.4 Samples for RNA sequencing and bioinformatics analysis

Samples for RNA extraction and sequencing were collected at true leaf‐age four. The third leaf and tissues near the shoot apical meristem (SAM, after removal of outside 2 layers of the sheath, keeping about ±3 mm of the growth point) were collected and put swiftly on ice and snap‐frozen in liquid N_2_, then stored at −80°C till use. Tillers were supposed to start to appear from the leaf axillary zone at this stage, therefore, the SAMs were collected for RNA sequencing to reflect tiller‐related gene profiles. RNA extraction, pre‐treatment, sequencing library generation, and bioinformatics algorithm all followed protocols of a previous report (Zhang et al., 2019). We chose four treatment‐level combinations (ambient‐N0, ambient‐N10, eCO_2_‐N0, and eCO_2_‐N10) for RNA sequencing analysis. Three independent biological replication samples (leaves or SAM were pooled from multiple plants) for each treatment combination were sequenced, and at least 6 Gb bases per sample were acquired, the minimum sample size needed for gene expression analysis (Table S1).

The filtered data (sequence excluding adaptor sequences used in cDNA library construction) were used for gene mapping and expression analysis following the protocol described in Trapnell et al., (2012). The FPKM (Fragments per kilo base of transcript per million mapped reads) value was used to indicate the expression level of a gene, which was calculated using this equation: total exon reads/(mapped reads (millions) × exon length (kilo base)). A differentially expressed gene (DEG) was defined as a gene having a average FPKM ratio between two treatment combinations greater than or equal to 2 (≥2, or ≤0.5) and with an adjusted false discovery rate (adj FDR) *p *≤ 0.05.

To validate the transcriptome data, 20 DEGs were selected and quantified using quantitative real‐time polymerase chain reaction (qRT‐PCR) following a previous protocol (Zhang et al., 2019). The qRT‐PCR quantification and FPKM expression data showed correlation coefficients of 0.632**~0.876**(*p *≤ 0.01), proving that the FPKM expression data were reliable.

### 2.5 Statistical analysis

The general linear model (GLM) procedure of SPSS (Ver22, IBM Inc.) was used to test for effects among treatments. For phenotypical and physiological index analyses, the CO_2_ level and N rate were deemed independent factors; for bioinformatics analysis, the CO_2_ level, N supply rate, and tissue type were defined as independent factors, and their full interactions were analyzed using the GLM procedure. The significance level was set at *p *≤ 0.05 for the least significant differences (LSD) *post hoc* test. Data presented in figures and tables were mean ± standard deviations.

## 3 RESULTS

### 3.1 Interactive effects of eCO_2_ and N application rate on rice tillering

**FIGURE 1:**
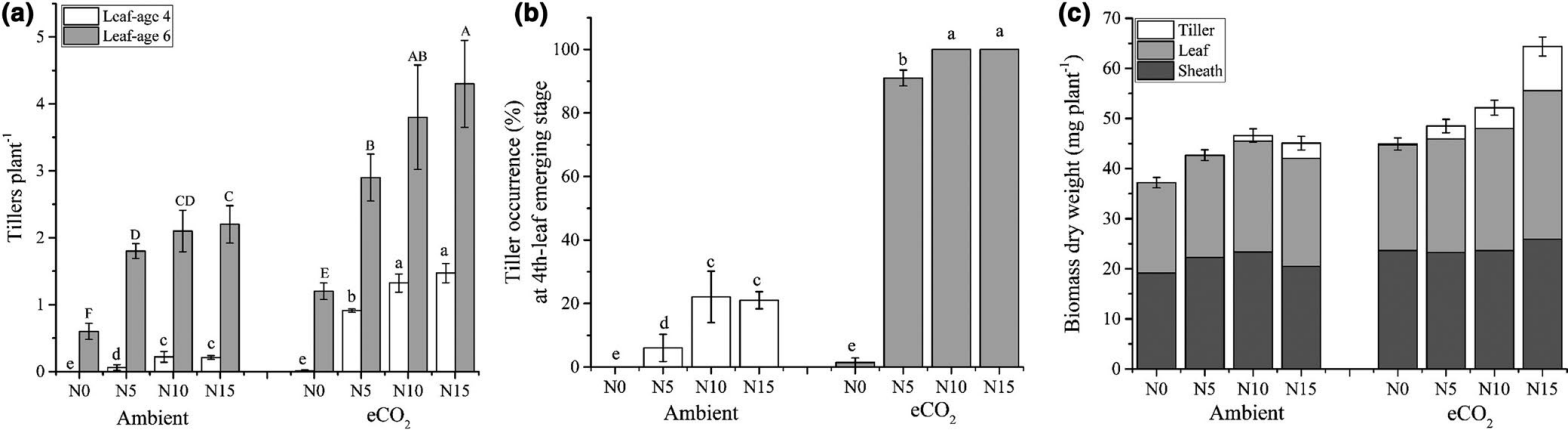
Tiller number at leaf‐age 4 and 6, tiller occurrence percentage, and biomass accumulation in response to N application rate and CO_2_ concentration. Amb and eCO_2_ stand for ambient and elevated CO_2_ treatment condition, respectively; N0, N5, N10, and N15 stand for N application rate 0, 75, 150, and 225 kg N ha^−1^, respectively; tiller number was the average of 192 plants (a); tiller occurrence percentage was investigated at leaf‐age 4 (b); biomass dry weight was sampled at leaf‐age 6, error bars are for the whole plant (c); error bars are standard deviations of four replicates; bars with different letters mean they are significantly different at *p* ≤ 0.05 by least significant differences comparison

Compared to the ambient [CO_2_], eCO_2_ significantly increased the number of tillers (Figure 1A, *p *≤ 0.01). At leaf‐age six, eCO_2_ promoted rice tillering by 0.6, 1.1, 1.7, and 2.1 tillers/plant at the corresponding N levels of N0, N5, N10, and N15 respectively. The number of tillers increased with the N application rate, with more significant enhancement at leaf‐age six than at leaf‐age four (*p *≤ 0.01). Under higher rates of N application, eCO_2_ stimulated more tillers (Figure 1A). Tiller occurrence percentage and uniformity were also enhanced by the eCO_2_ at the tiller starting stage (Figure 1B), which increased from 0, 6.0, 22.1, and 21.0% under the control [CO_2_] to 1.43, 91.0, 100, and 100% under the eCO_2_ at the corresponding N0, N5, N10, and N15 treatments. Apparently, the tiller enhancement effect of eCO_2_ depended on the N application rate, confirming that N availability constrained tillering, especially under eCO_2_.

Similarly, eCO_2_ increased biomass by 24.5, 13.8, 11.8, and 42.6% at the corresponding N0, N5, and N15 treatments (Figure 1C). Though N application promoted biomass accumulation under both ambient and eCO_2_ conditions, biomass accumulation reached a plateau at the N10 treatment under ambient [CO_2_]. In contrast, at the N15 treatment, biomass still showed a significant increase over that of N10 under eCO_2_ (Figure 1C, *p* ≤ 0.05). It can be concluded that eCO_2_ promoted biomass accumulation, especially when combined with a higher N application rate.

### 3.2 Relative change in allocation of N to newly emerged tillers under eCO_2_

**FIGURE 2:**
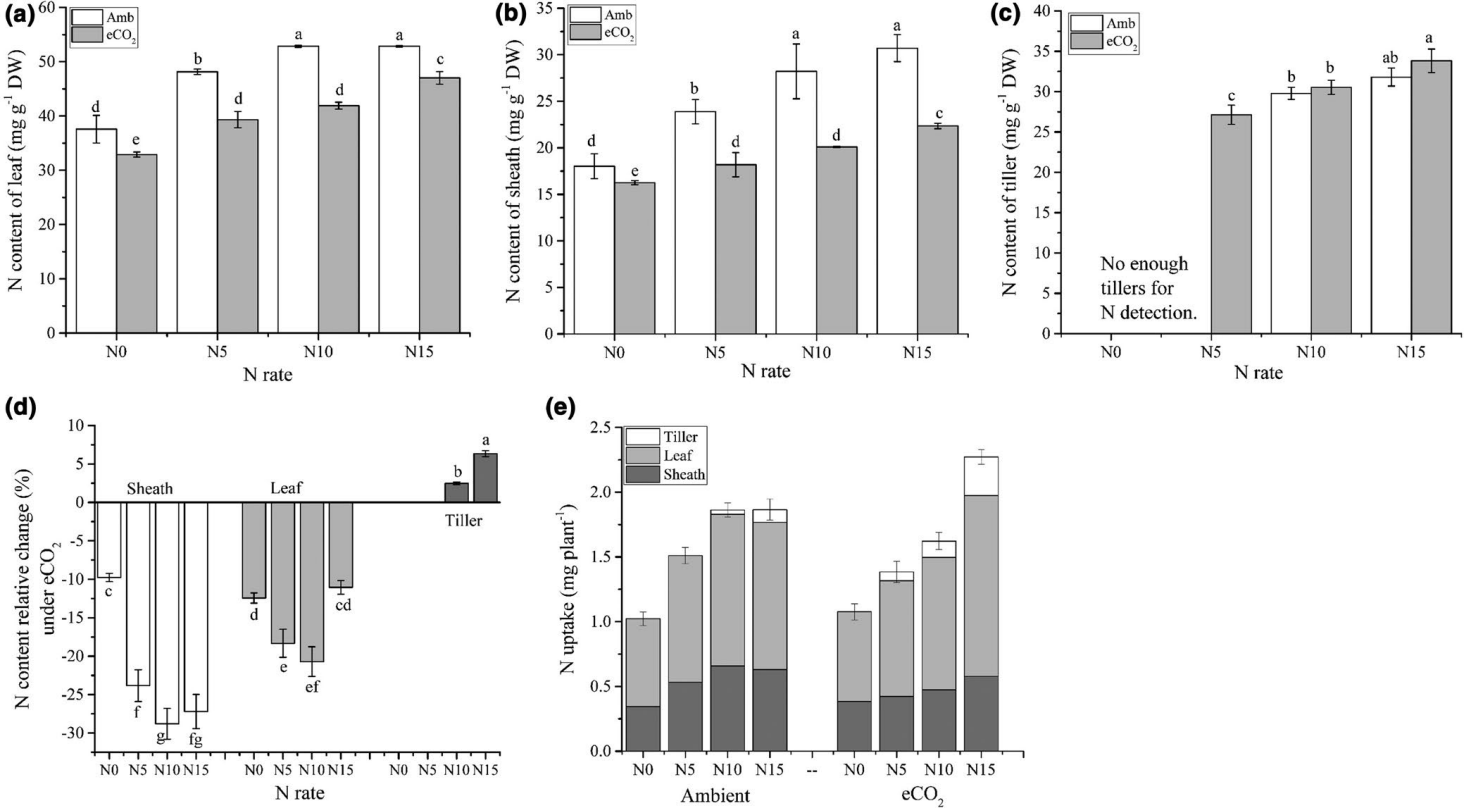
The responses of N content in leaf, sheath, and newly emerged tillers to N application rate and CO_2_ conditions (a, b, and c), N uptake (d), and their relative change under eCO_2_ to ambient CO_2_ condition (e). Treatment information is the same as in Figure 1; N content was from tissues harvested at leaf‐age 6; error bars are standard deviations; bars with different letters mean they are significantly different at *p* ≤ 0.05 by least significant differences comparison

Higher N application rate increased N content in both leaf and sheath, regardless of CO_2_ treatment (Figure 2A and B). However, eCO_2_ reduced the N content consistently in all N application rates, compared to the ambient CO_2_ treatments (*p *≤ 0.01). It is worth mentioning that under ambient [CO_2_], N content reached a plateau in the N10 treatment, whereas under the eCO_2_ it still increased at the N15 treatment, regardless of tissue type (leaf or sheath). The N uptake per plant at the leaf‐age six was lower under the eCO_2_ than under the ambient [CO_2_], except in the N15 treatment (Figure 2C), mostly due to the reduction in N content because biomass increased slightly. This further showed that the N application rate was more of a constraint to N uptake under the eCO_2_ than under ambient [CO_2_].

Contrary with the reductions in N content in the leaves and sheaths of rice plants exposed to eCO_2_, the new‐born tillers of plants exposed to eCO_2_ tended to have slightly higher N content than tillers produced at ambient [CO_2_] (Figure 2D). Newly emerged tillers under eCO_2_ showed slight increases (2.5 and 6.4 mg g^−1^ at the N10 and N15 treatments, respectively, Figure 2E). A plausible explanation is that rice plants allocated a higher proportion of N to newly emerged tillers when exposed to eCO_2_.

### 3.3 Carbohydrate accumulation promoted by the eCO_2_

**FIGURE 3:**
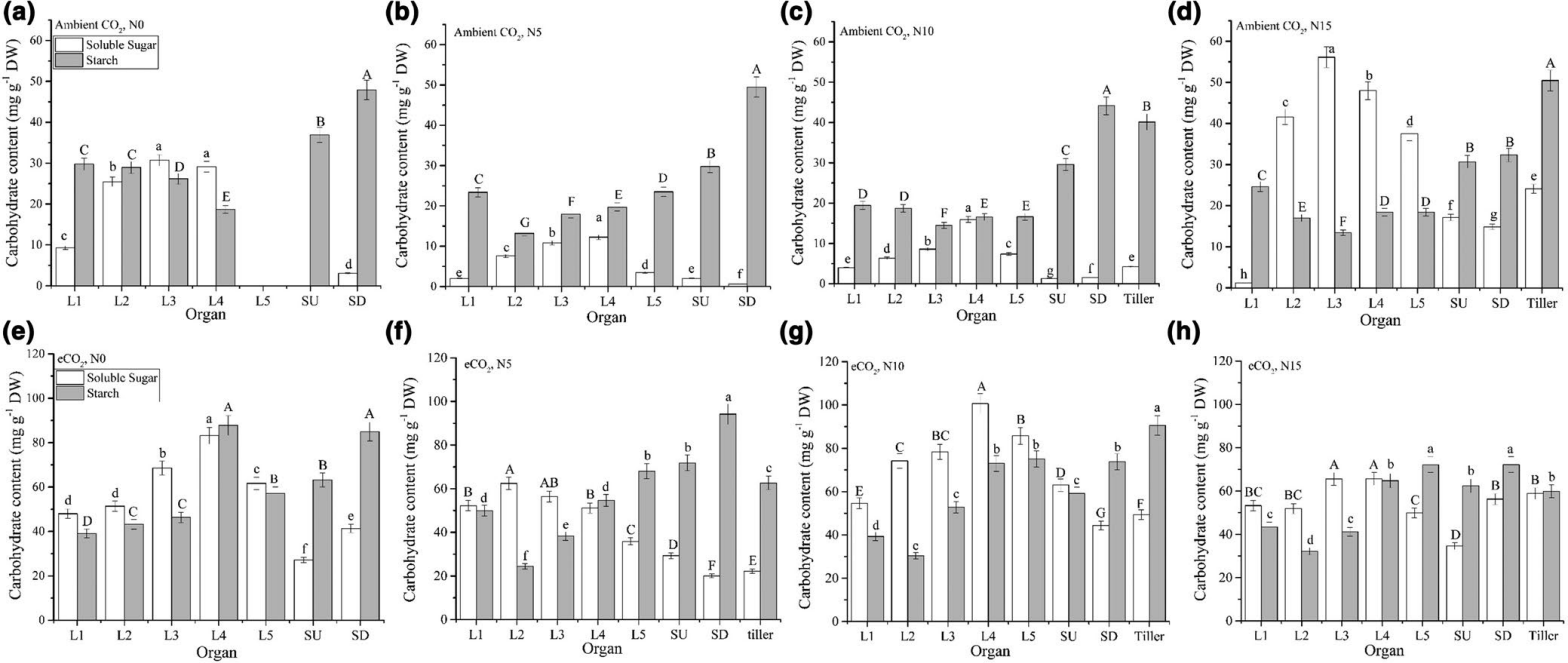
Carbohydrates change in response to N application rate and CO_2_ treatment. Treatment information is the same as in Figure 1; tissues were harvested at leaf‐age 6; L1‐L5 represent leaf positions 1–5 of the main stem (counted from bottom up), respectively; SU and SD represent up and down part of the sheath, respectively; tiller were newly emerged tillers at leaf‐age 6; significant differences were detected in every corresponding counterpart between ambient and eCO_2_ treatments; error bars are standard deviations; bars with different letters mean they are significantly different at *p* ≤ 0.05 by least significant differences comparison, small and capital letters are for starch and soluble sugar contents respectively

Under ambient [CO_2_], soluble sugar content was significantly reduced when the N application rate increased from N0 to N5 and N10, dropping from 16.3 to 5.5 and 6.2 mg g^−1^ (comparison of Figure 3B, 3C to 3A, *p* ≤ 0.01). Further N enrichment to N15 reversed this trend and significantly increased soluble sugar content to 30.1 mg g^−1^ (Figure 3D, *p* ≤ 0.05). However, starch content changed relatively at small scales from 31.4 to 25.3, 25.0, and 25.7 mg g^−1^ at the N0, N5, N10, and N15 treatments (Figure 3A–D). Likewise, under eCO_2_, enrichment of N from the N0 to N5 reduced soluble sugar (being 54.5 to 41.2 mg g^−1^), reaching a peak at the N10 (68.8 mg g^−1^), but further N enrichment to the N15 reduced soluble sugar content (54.5 mg g^−1^). In contrast, the starch content under the eCO_2_ was consistently high under all N application rates, being 60.3, 58.0, 61.8, and 56.0 mg g^−1^, respectively (Figure 3E–H). The newly emerged tillers under the eCO_2_ had higher contents of both soluble sugar and starch than under the ambient CO_2_, implying that relatively higher distribution of N content in them was not a result of carbohydrate drainage (Figure 3C, D vs. G, H). Overall, under the eCO_2_ condition, all tissues had higher carbohydrate content than under the ambient CO_2_, regardless of N application rate.

### 3.4 Limited global transcriptomic change induced by eCO_2_ than by N application

**FIGURE 4:**
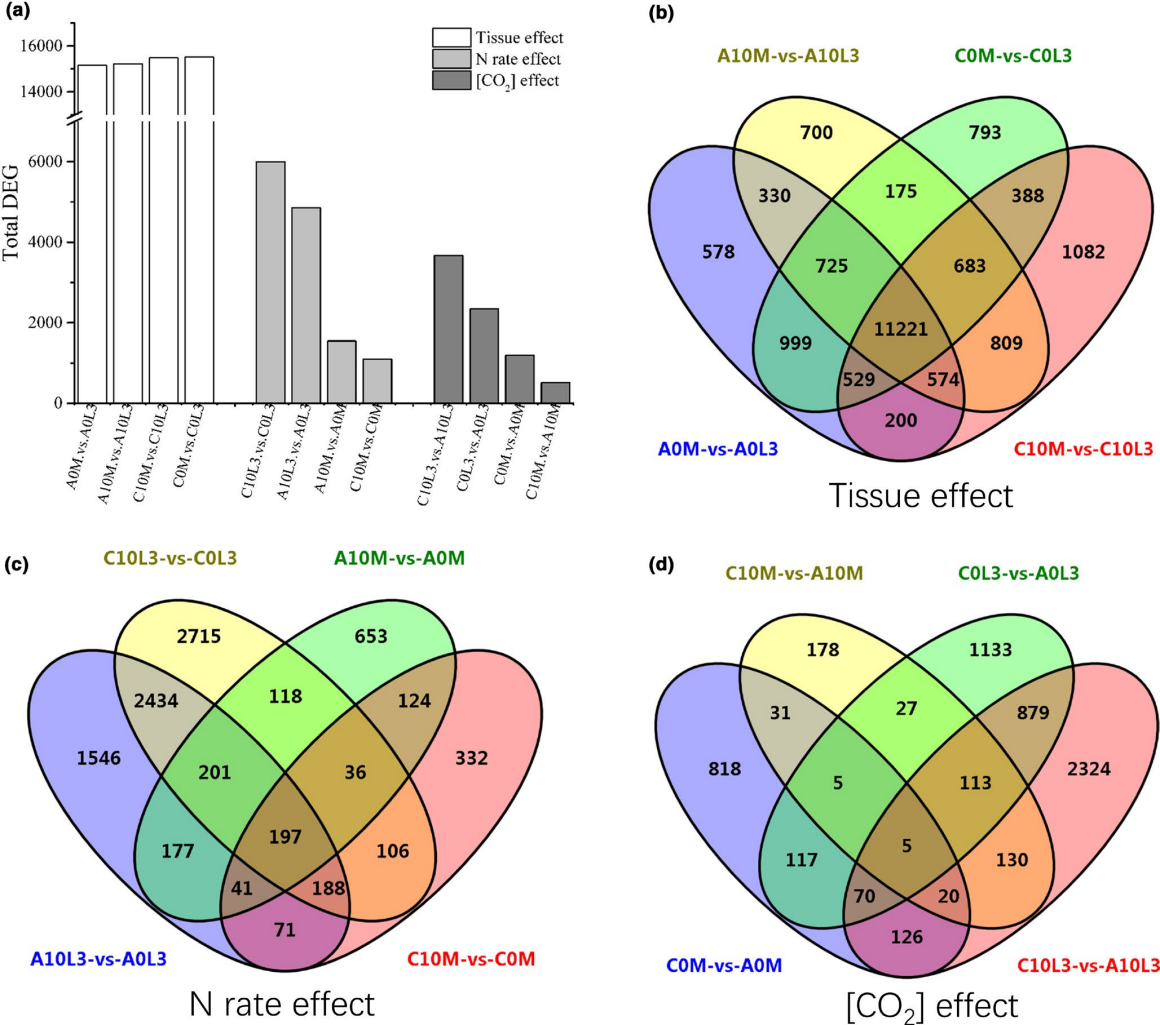
Histogram of the number of differentially expressed genes (DEG) and Venn diagrams of comparisons of tissue type, CO_2_, and N application effects. In the labels for each oval, the CO_2_ treatment levels, which come first, are A (ambient) or C (eCO_2_), the nitrogen treatment levels, in the second position, are 0 (N0) or 10 (N10), and, in the last position, L3 (leaf number 3) or M (apical meristem) represent samples from different tissue types

To explore the underlying transcriptional changes in response to eCO_2_, we employed an RNA sequencing approach to compare gene expression profiles of tissues near the SAM (where the tiller was about to appear) and the third true leaf at leaf‐age four. The total numbers of DEG found were dramatically different in different tissue types (leaf and SAM), N application rates (N0 and N10), and CO_2_ treatment levels (ambient CO_2_ and eCO_2_) (Figure 4A). Tissue type had the largest effect (15156~15513 DEG), followed by N application rate (1095~5995 DEG), and eCO_2_ (509~3667 DEG). Both N application rate and CO_2_ level had more influence on leaf DEG number than on SAM DEG number (Figure 4A).

Using Venn diagrams to illustrate tissue, CO_2_ level, and nitrogen treatment effects on differential gene expression, we found that the universal number of DEG (commonly altered genes) between tissue types was greatest (11221, Figure 4B), followed by N application (197, Figure 4C), and lastly by CO_2_ (5, Figure 4D). These numbers indicated that the global gene expression profile was overwhelmingly defined by the tissue type, only being altered at a limited range by N application rate, and at a very narrow scale by eCO_2_.

### 3.5 CO_2_ and N application effects on expression of tiller‐related genes

To elaborate the responses of tiller‐related genes to eCO_2_ and N application rate, we extracted transcription data of 65 tiller‐related genes from the transcriptome data. Among them, 51 genes were expressed (averagely FPKM> 0.1) in leaf and/or SAM at the 4th true leaf emerging stage. Fifty of those 51 genes’ expression varied significantly between the tissues (leaf and SAM, *p* ≤ 0.05), 29 of the 51 genes’ expression were altered significantly by [CO_2_], and 24 of the 51 gene's expression were significantly affected by N application (Table S2, *p* ≤ 0.05). This further supports the conclusion that tissue type (organ) predominantly defines the gene expression profile.

However, as more tiller genes responded to [CO_2_] than to N application rate (Table 1), which was unexpected given that the opposite held for the overall transcriptome data (Figure 4A and 4B). Of the 29 [CO_2_] responsive tiller‐genes and 24 N application responsive tiller‐genes, 16 genes responded to both factors, 13 responded to [CO_2_] but not to N application, and only 8 responded to N application but not [CO_2_], once again, contrary with their differential influences on the global transcriptomic profile.

**TABLE 1:** The relative FPKM change of tiller‐related genes in response to tissue type, [CO_2_] and N rate

Gene name	LOC_ID#	Ambient		eCO_2_		Leaf		SAM	
		Leaf	SAM	Leaf	SAM	N0	N10	N0	N10
		N10/N0	N10/N0	N10/N0	N10/N0	eCO_2_/Amb	eCO_2_/Amb	eCO_2_/Amb	eCO_2_/Amb
RFL	Os04g51000		0.64		1.13			0.45	0.80
OsRPK1	Os05g40770	3.46	0.15	5.98	1.03	2.49	4.29	0.85	5.73
DLT	Os06g03710	2.45	0.60	1.21	0.85	3.95	1.94	0.72	1.01
BES1/BZR1/OsBZR1	Os07g39220	0.94	0.69	1.10	1.00	1.42	1.66	0.72	1.03
D11	Os04g39430	0.70	0.75	1.74	0.87	0.43	1.07	0.82	0.95
CGMC_GSK.4	Os02g14130	0.86	0.90	0.94	0.97	0.87	0.94	0.97	1.04
D2	Os01g10040	0.36	0.42	0.75	1.42	0.59	1.22	0.48	1.64
TAL	Os01g70170	2.15	1.35	4.36	1.42	0.79	1.61	1.03	1.09
LAX1	Os01g61480		1.55		1.04			1.54	1.04
BRD1	Os03g40540	0.80	1.33	0.81	1.11	0.90	0.91	1.27	1.06
OsSPL14/IPA1/WFP	Os08g39890	1.28	1.44	1.21	1.24	2.08	1.97	1.23	1.06
qPN1	Os01g70550	1.62	1.17	1.80	1.44	0.66	0.73	0.94	1.16
OsNAC2	Os08g06140	2.11	0.97	1.10	0.97	2.82	1.47	1.02	1.03
GID2/14‐3‐3	Os02g36974	1.00	1.04	1.33	0.99	0.99	1.32	1.04	1.00
slr1	Os03g49990	1.58	1.02	1.33	1.23	0.91	0.76	0.85	1.03
sdg/GID1	Os05g33730	0.92	0.79	1.16	0.80	1.28	1.61	1.11	1.11
MOC1	Os06g40780	2.52	0.53	1.39	0.81	1.12	0.62	0.38	0.58
OsWOX4	Os04g55590		1.12		0.82			1.62	1.19
Lazy1 (La1)	Os11g29840	0.53	0.64	3.59	1.49	0.16	1.09	0.41	0.96
D14/D88/HTD2	Os03g10620	0.78	0.50	2.04	1.95	0.22	0.56	0.35	1.37
D3/OsFBL27 /AtMAX2/AtOER9	Os06g06050	0.71	0.86	1.31	1.17	0.36	0.66	0.82	1.12
LIC	Os06g49080	1.24	1.07	0.87	0.91	0.88	0.62	1.00	0.86
OsEXP4	Os05g39990	0.43	0.72	2.29	1.21	0.38	2.01	0.65	1.09
BRI1/D61	Os01g52050	1.65	1.02	1.27	0.89	1.49	1.14	1.13	0.98
D10	Os01g54270	1.43	0.90	3.29	1.20	0.35	0.80	1.23	1.64
OsPIN1	Os02g07630	0.96	0.95	0.95	0.99	0.86	0.84	0.98	1.02
OsBIN2	Os01g10840	1.12	0.99	1.36	0.91	0.88	1.07	1.09	1.01
APC/C(TE)	Os03g03150	1.06	1.07	1.35	0.88	1.10	1.40	1.12	0.92
CRCT	Os05g51690	1.14	0.97	0.59	1.14	1.99	1.04	0.96	1.13
D17/HTD1	Os04g46470	0.48	0.69	0.97	0.89	0.86	1.74	0.80	1.04
BAK1	Os08g07760	0.95	0.86	1.03	0.95	1.07	1.17	0.91	1.01
OsCDC27	Os06g41750	1.44	0.98	1.69	0.99	1.00	1.17	0.94	0.94
RCN1/OsABCG5	Os03g17350	1.88	1.55	3.82	1.91	0.71	1.45	1.08	1.33
OsHAP2E	Os03g29760	1.95	0.95	1.53	1.09	1.18	0.93	0.90	1.03
IBH1/OsIBH1	Os04g56500	0.55	0.32	0.77	1.14	0.86	1.22	0.73	2.63
SD1	Os01g66100	0.22	0.56	0.09	0.90	0.73	0.32	0.81	1.31
CGMC_GSK.8	Os06g35530	1.31	1.13	1.44	1.12	0.98	1.08	1.04	1.02
LAX2	Os04g32510	0.39	0.65	0.94	0.92	2.22	5.36	0.40	0.57
OsMADS57	Os02g49480	0.67	1.08	6.19	0.80	3.19	29.26	1.07	0.79
LRK1	Os02g05980	1.10	0.64	0.90	1.79	0.98	0.81	0.67	1.87
ILI1	Os04g54900		1.56					0.00	0.68
GDH7	Os07g15770	0.47		1.04		0.66	1.45		
OsPIN2	Os06g44970		0.96		0.97			0.76	0.77
BKI1	Os09g28550	0.86	0.62	1.28	0.67	0.75	1.11	0.98	1.07
OsTB1/fc1	Os03g49880	0.16	0.90		1.23	0.00	1.04	0.69	0.95
GA2ox1	Os05g06670		1.14	1.04	1.11			1.12	1.08
OsAPC10	Os05g50360	0.90	1.02	1.09	0.95	0.95	1.16	0.92	0.86
D63	Os08g01110	0.93	1.08	0.99	0.98	0.85	0.91	1.04	0.94
DEP1	Os09g26999	2.34	1.10	2.29	1.06	2.06	2.02	0.93	0.89
OsFEN‐1	Os05g46270	1.03	1.05	2.16	0.96	0.41	0.85	1.05	0.97
OsH1	Os03g51690	5.71	1.06	2.38	0.97	6.16	2.57	0.99	0.91

Note:

Red and blue text denote up or down‐regulated fold change surpass twofold to be DEG respectively. SAM stands for shoot apical meristem. Ambient and eCO_2_ are for ambient and elevated CO_2_ concentration respectively. N0 and N10 are for N application rate 0 and 10, equal to 0, and 150 Kg N ha^−1^ respectively.

For these tiller genes, N application caused a more dramatic change in gene expression the leaf than in the SAM. In the leaf, 14 (7 up, 7 down, for ambient CO_2_) and 12 (11 up, 1 down, for e CO_2_) tiller genes surpassed the twofold threshold to count as DEG. In the SAM, these numbers were three (all down) and 0 for the ambient CO_2_ and eCO_2_ respectively. In similar fashion, [CO_2_] effects in the leaf were 16 (8 up and 8 down, for the N0 treatment level) and seven (all down, for the N10 treatment level) tiller‐genes surpassed two‐fold changes in expression, while these values were seven (6 up and 1 down, for N0) and two (up, for N10) in SAM. The fact that most tiller genes’ expression was altered in the leaf rather than in the SAM indicated that either most of them were not participating in the tillering response or that the scale of change did not often surpass the arbitrarily chosen twofold threshold. It is also possible that the genes affecting tillering‐promotion most affected by altered [CO_2_] were not among the 65 extracted genes from the transcriptome.

### 3.6 N transporters and glutamate receptors were differentially altered in the leaf and SAM

**FIGURE 5:**
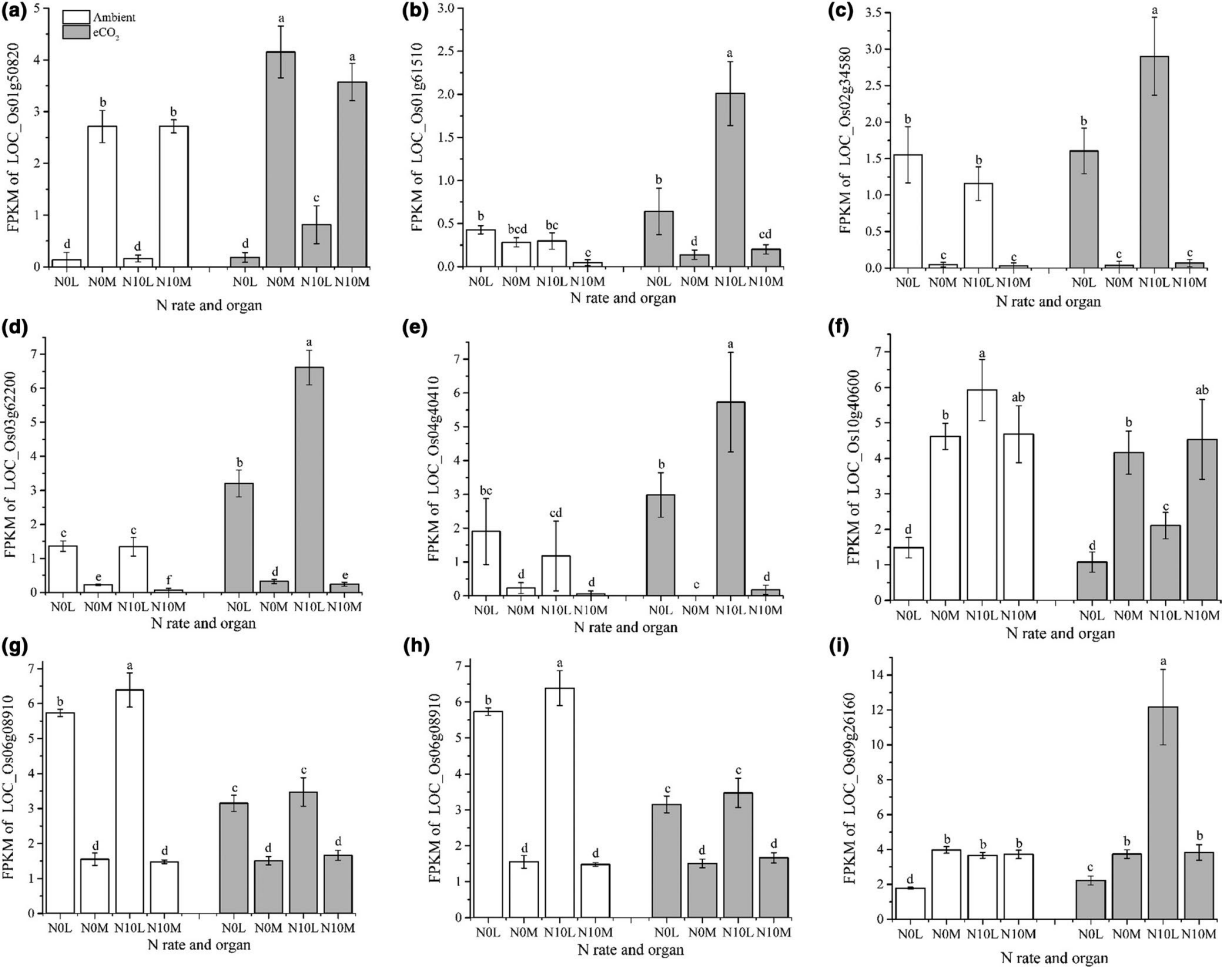
The gene expression (FPKM) in leaf and SAM in response to N application and CO_2_ condition. L for leaf, M for shoot apical meristem; Ambient and eCO_2_ stand for ambient and enriched CO_2_ treatments, respectively; error bars are standard deviations; bars with different letters mean they are significantly different at *p* ≤ 0.05 by least significant differences comparison

Since N application rate dramatically changed gene expression, we screened the transcriptome dataset for genes involved in N metabolism. Of 210 N‐metabolism related genes, 164 had altered expression (average FPKM> 0.1). Among them, 158 genes were significantly impacted by tissue type, 109 genes were affected by N application rate, and 94 were affected by [CO_2_] (Table S3, *p *≤ 0.05). Not surprisingly, eCO_2_ exerted less influence on this set of genes than N application did. Examination of the putative functions of the 94 genes significantly affected by [CO_2_] had revealed that 20 (21.3%) were glutamate receptors or nitrate or ammonium transporters. In contrast, only seven of the 70 (10%) remaining genes that did not respond to changes in [CO_2_] non‐responsive genes (not significant, *p *≥ 0.05) were glutamate receptors or nitrate or ammonium transporters. It seems that more N‐metabolism receptors and transporters were altered by eCO_2_, suggesting that N redistribution/reallocation may become more active under eCO2. Among them, nine genes displayed contrasting induction and suppression changes between the leaf and SAM (Figure 5). They were putative major facilitator family transporter (NRT2.3a/b, nitrate transporter, LOC_Os01g50820, Figure 5A), ammonium transporters (LOC_Os01g61510, LOC_Os02g34580, and LOC_Os03g62200, Figure 5B–D), high‐affinity nitrate transporter (LOC_Os04g40410, Figure 5E), and peptide transporter PTR2 (NRT1.1a/b, LOC_Os10g40600, Figure 5F). The others were all putative glutamate receptors (LOC_Os06g08910, LOC_Os09g26144, and LOC_Os09g26160, Figure 5G–H). These genes were likely the critical contributors to the change of N distribution among organs in the eCO_2_ adaptation process in rice plants.

## 4 DISCUSSION

Significant effects of [CO_2_], N application, and their interaction on tillering were found in this study (Figure 1). N enrichment promoted tillering under both ambient CO_2_ and eCO_2_ confirming that N availability is a constraint to rice tillering. However, the N promotion effect on tillering diminished at the N10 level under ambient CO_2_, but remained even at the N15 under eCO_2_. These responses suggested that N’s limitation to tillering was aggravated under eCO_2_. This was further corroborated by the decreased N content in leaf and sheath and the increased carbohydrates under eCO_2_. This implies that more N input will be needed to take advantage of the CO_2_ fertilization effect under future eCO_2_ conditions in order to attain more tillers. Newly emerged tillers actually had comparable or higher N contents under eCO_2_ when compared to ambient CO_2_ (Figure 2D), whereas leaf and sheath showed a significant decline in N content. The more dramatic decline of N content in the sheath than in the leaf was consistent with previous findings (Huang et al., 2004; Jitla et al., 1997). Therefore, there could be a reallocation of N toward the SAM under eCO_2_ (newly emerged tillers include novel SAM). Unfortunately, we did not investigate the N content of root tissue in this experiment. Our previous FACE experiments demonstrated that roots also display a reduction of N content in percentage under eCO_2_ (Huang et al., 2004). As the N constraint is persistent in an unmanaged niche (Mueller et al., 2013; Reich et al., 2006), plants likely adapt to distribute more of their acquired N to areas where cell are proliferating rather than maintaining the original distribution pattern. For this reason, future rice production may require more N input to take advantage of eCO_2_, although the amount may vary in different species and varieties.

To reveal the molecular mechanism underlying this change, we conducted RNA‐sequencing to obtain a complete profile of transcriptomic alteration between the SAM and leaf tissues under four combinations of [CO_2_] levels and N application rates (Figure 4). Though tissue differences predominantly defined changes in the transcription profile globally, N application generally had a greater effect than altered [CO_2_]. This is understandable as N has been proved to be a major limiting factor in plant growth (Xuan et al., 2017; Zhang et al., 2019). Expression alterations for a group of N metabolism‐related genes were consistent with global transcriptomic differences, i.e., N application overrode the influence of altered [CO_2_]. However, when tiller‐related genes were examined, they were influenced more by altered [CO_2_] than by changes in N application. We speculate that the redistribution of N favoring the SAM under eCO_2_ magnified the impact of changed [CO_2_] on tiller‐related gene expression.

Examination of the putative functions of those N metabolism‐related genes with expression altered under eCO_2_ revealed that a portion of N receptors and transporters were down‐regulated in both leaf and SAM tissue, including LOC_Os10g40600 (Figure 5F) encoded OsNRT1.1a/b, which has been shown to function in nitrate sensing, transport, and remobilization (Fan et al., 2016; Hu et al., 2015; Wang, Hu, et al., 2018). Other studies suggest that there might be other genes that regulate the N signaling and transportation (Feng et al., 2011; Wang et al., 2019) in response to eCO_2_. As shown here, *OsNRT2*.*3a*/*b* (LOC_Os01g50820, Figure 5A) had a similar expression level (FPKM) to *OsNRT1*.*1a*/*b*, and two glutamate receptors (LOC_Os06g08910 and LOC_Os09g26160, Figure 5G and I, respectively) also showed significant expression alterations in both leaf and SAM, which were differentially regulated in those tissues under altered [CO_2_] (Figure 5). This suggests that those loci were coordinately regulated in the process of eCO_2_ adaptation. There is an integrative co‐expression network that determines N use efficiency (Zhang et al., 2019). Exploitation of this interconnectivity may mean that alteration of one target gene could potentially increase N use efficiency and crop yield (Xu et al., 2019; Zeng et al., 2017). Stacking (pyramiding) more target loci together, especially loci in certain important network pathways, may eventually create a substantial yield breakthrough. Our results here provided a list of potential targets for such an approach.

Knowledge of the underlying mechanism of eCO_2_ adaptation in crops can provide us useful information for breeders involved in genetic engineering (Ainsworth & Ort, 2010; Fletcher, 2018; Schimel, 2006). Our preliminary results demonstrate that the N sensor and transporter genes played important roles in responses to eCO_2_. We believe that this understanding might be extended to some other plants, as under eCO_2_, elemental stoichiometry reveals a generally consistent reduction of N content in most terrestrial plants, but their growth and biomass accumulation are accelerated to a variating degree (Deng et al., 2015; Norby et al., 2010; Reich et al., 2006). The plausible reason lies in the fact that, in plants, redistribution of acquired N to cell‐division zones such as SAM and new‐branches differs from changes found in other tissues. This might be an adaptation strategy in most terrestrial plants. Consequently, genetic engineering focused on these loci that facilitate N redistribution may improve CO_2_ fixation rate and eventually biomass accumulation and economic yield in those plants.

In summary, eCO_2_ increased carbohydrates in all examined rice plant organs and lowered N contents in leaves and sheath as expected. However, newly emerged tillers maintained N content comparable to or higher than that under the control CO_2_ condition. The N redistribution among the organs was likely accomplished by coordinated regulation of N metabolism receptor and transporter gene expression in both leaf and SAM.

## 5 CONFLICT OF INTEREST STATEMENT

All authors have no conflict of interest to declare.

## AUTHOR CONTRIBUTIONS

JZ, YY, JH, and YW: Conceptualization; JZ, YG, JW, ZW, ML, CC, YZ, XZ, and GD: Investigation, Data curation and Analysis, Visualization and Validation; JZ and YY: Original draft; YY, DH, and ZY: Review and Editing.

## Supporting information

Table S1‐S3
