# Supplementary material for: Elevated atmospheric CO2 concentration triggers redistribution of nitrogen to promote tillering in rice
Source: Plant Environ Interact. 2021 May 8;2(3):125–36. doi: 10.1002/pei3.10046 (PMC10168068; doi:10.1002/pei3.10046)
Supplement: Supplementary file 1 — Table S1‐S3 [file PEI3-2-125-s001.pdf]

Table S1 Treatment samples for RNA sequencing and the generated data information and qualification

[CO_2]	N rate	Tissue	rep	raw reads	raw bases	clean reads	clean bases	valid bases	Q30	GC	Clean reads%
Ambient	0	Leaf3	1	41637708	6.246E+09	39801920	5966063267	95.52%	92.43%	49.50%	95.59%
Ambient	0	Leaf3	2	44697614	6.705E+09	42376046	6351405081	94.73%	91.76%	50.50%	94.81%
Ambient	0	Leaf3	3	44125088	6.619E+09	41904002	6280677253	94.89%	91.65%	51.00%	94.97%
Ambient	0	SAM	1	50698254	7.605E+09	48148120	7216764178	94.89%	91.86%	49.50%	94.97%
Ambient	0	SAM	2	42004460	6.301E+09	39893938	5979595203	94.90%	91.89%	49.50%	94.98%
Ambient	0	SAM	3	45231516	6.785E+09	42927908	6434391752	94.83%	91.93%	49.00%	94.91%
Ambient	10	Leaf3	1	51745006	7.762E+09	50628638	7589464940	97.78%	94.08%	49.50%	97.84%
Ambient	10	Leaf3	2	51159050	7.674E+09	49775406	7461289705	97.22%	93.57%	49.00%	97.30%
Ambient	10	Leaf3	3	50895730	7.634E+09	49669028	7445486788	97.52%	93.84%	49.50%	97.59%
Ambient	10	SAM	1	51247446	7.687E+09	50005324	7496134277	97.51%	93.94%	47.50%	97.58%
Ambient	10	SAM	2	52732628	7.91E+09	51624968	7738790253	97.83%	93.92%	48.00%	97.90%
Ambient	10	SAM	3	51753798	7.763E+09	50776742	7611916033	98.05%	94.23%	47.50%	98.11%
eCO2	0	Leaf3	1	39445000	5.917E+09	37451368	5613326981	94.87%	91.83%	50.50%	94.95%
eCO2	0	Leaf3	2	45434090	6.815E+09	43383470	6502804730	95.41%	92.37%	49.50%	95.49%
eCO2	0	Leaf3	3	51715438	7.757E+09	50636584	7590805833	97.85%	94.24%	49.50%	97.91%
eCO2	0	SAM	1	52640338	7.896E+09	51556796	7728877480	97.88%	94.35%	47.50%	97.94%
eCO2	0	SAM	2	53685778	8.053E+09	52459688	7864120699	97.65%	94.06%	47.50%	97.72%
eCO2	0	SAM	3	52357906	7.854E+09	51303392	7690801923	97.92%	94.34%	47.50%	97.99%
eCO2	10	Leaf3	1	52967094	7.945E+09	51499294	7719435421	97.16%	93.16%	49.50%	97.23%
eCO2	10	Leaf3	2	51775558	7.766E+09	50615368	7587298391	97.69%	93.73%	48.50%	97.76%
eCO2	10	Leaf3	3	50841836	7.626E+09	49657890	7443775418	97.60%	93.69%	49.50%	97.67%
eCO2	10	SAM	1	51566014	7.735E+09	50368754	7550489262	97.61%	93.75%	47.50%	97.68%
eCO2	10	SAM	2	51996872	7.8E+09	50932032	7635073435	97.89%	94.06%	48.50%	97.95%
eCO2	10	SAM	3	53638896	8.046E+09	52424600	7858658491	97.67%	93.78%	48.50%	97.74%

Ambient and eCO_2 stand for ambient and enriched CO_2 concentration, respectively; N rate 0 and 10 stand for N application rate (kg N 666.67 m^{-2}), respectively; SAM stands for shoot apical meristem, and leaf3 for the third leaf from bottom at leaf age 4 growth stage; rep: replicate; raw reads: original number of reads from high throughput sequencing Illumina IIx; raw bases: the base pair number of the raw reads; clean reads: filtered reads after removing adptor or invalid sequences; clean bases: the base number of clead reads; valid bases: the base number of clean reads/base number of raw base reads *100%; Q30: the percentage of quality score that meet the probability of correct call of base was greater than 99.9%; GC: the overall G+C% of the clean reads, AT=100-GC; clean reads% equals clean reads/raw reads * 100%.

Table S2. The significance level of tiller-related gene affected by [CO_2], N rate and organ.

Gene name	LOC_ID#	Significancy level at *P* value						
		[CO_2]	N rate	Organ	[CO_2]*N rate	[CO_2] * Organ	N * Organ	[CO_2] * N * Organ
RFL	Os04g51000	0.000	0.034	0.000	0.005	0.000	0.030	0.005
OsRPK1	Os05g40770	0.000	0.000	0.000	0.001	0.000	0.000	0.001
DLT	Os06g03710	0.000	0.000	0.000	0.000	0.000	0.000	0.000
BES1/BZR1/OsBZR1	Os07g39220	0.015	0.001	0.000	0.000	0.000	0.001	0.001
D11	Os04g39430	0.001	0.000	0.000	0.008	0.028	0.000	0.280
CGMC_GSK.4	Os02g14130	0.034	0.000	0.000	0.005	0.046	0.090	0.249
D2	Os01g10040	0.003	0.000	0.000	0.000	0.052	0.019	0.000
TAL	Os01g70170	0.003	0.000	0.000	0.004	0.950	0.014	0.198
LAX1	Os01g61480	0.014	0.013	0.000	0.058	0.021	0.020	0.039
BRD1	Os03g40540	0.022	0.005	0.000	0.157	0.006	0.000	0.133
OsSPL14/IPA1/WFP	Os08g39890	0.015	0.000	0.000	0.206	0.016	0.000	0.205
qPN1	Os01g70550	0.000	0.000	0.000	0.636	0.000	0.000	0.388
OsNAC2	Os08g06140	0.000	0.028	0.000	0.124	0.000	0.006	0.105
GID2/14-3-3	Os02g36974	0.011	0.013	0.000	0.119	0.125	0.094	0.009
slr1	Os03g49990	0.037	0.000	0.000	0.114	0.565	0.270	0.015
sdg/GID1	Os05g33730	0.007	0.006	0.000	0.742	0.898	0.002	0.517
MOC1	Os06g40780	0.015	0.676	0.016	0.624	0.087	0.024	0.118
OsWOX4	Os04g55590	0.000	0.293	0.000	0.019	0.000	0.259	0.016
Lazy1 (La1)	Os11g29840	0.000	0.147	0.000	0.000	0.000	0.153	0.000
D14/D88/HTD2	Os03g10620	0.000	0.906	0.000	0.000	0.000	0.888	0.008
D3/OsFBL27	Os06g06050	0.000	0.227	0.038	0.001	0.000	0.227	0.231
LIC	Os06g49080	0.002	0.741	0.000	0.012	0.407	0.541	0.903
OsEXP4	Os05g39990	0.047	0.307	0.000	0.006	0.051	0.314	0.009
BRI1/D61	Os01g52050	0.002	0.263	0.000	0.001	0.090	0.002	0.005
D10	Os01g54270	0.006	0.534	0.000	0.181	0.005	0.623	0.192
OsPIN1	Os02g07630	0.036	0.181	0.000	0.661	0.030	0.963	0.575
OsBIN2	Os01g10840	0.045	0.414	0.000	0.215	0.025	0.001	0.012
APC/C(TE)	Os03g03150	0.010	0.236	0.000	0.239	0.059	0.008	0.000
CRCT	Os05g51690	0.003	0.119	0.361	0.040	0.012	0.027	0.003
D17/HTD1	Os04g46470	0.069	0.000	0.000	0.004	0.027	0.000	0.033
BAK1	Os08g07760	0.356	0.000	0.000	0.015	0.005	0.001	0.079
OsCDC27	Os06g41750	0.443	0.002	0.000	0.354	0.018	0.000	0.403
RCN1/OsABCG5	Os03g17350	0.089	0.000	0.000	0.161	0.140	0.002	0.356
OsHAP2E	Os03g29760	0.737	0.001	0.000	0.862	0.564	0.003	0.112
IBH1	Os04g56500	0.862	0.016	0.000	0.126	0.760	0.133	0.927
SD1	Os01g66100	0.260	0.000	0.000	0.078	0.323	0.137	0.235
CGMC_GSK.8	Os06g35530	0.179	0.000	0.000	0.692	0.500	0.407	0.489
LAX2	Os04g32510	0.052	0.247	0.000	0.343	0.009	0.339	0.414
OsMADS57	Os02g49480	0.724	0.829	0.000	0.501	0.047	0.108	0.016

LRK1	Os02g05980	0.152	0.816	0.000	0.753	0.050	0.830	0.014
ILI1	Os04g54900	0.336	0.301	0.030	0.749	0.336	0.301	0.749
GDH7	Os07g15770	0.817	0.357	0.000	0.312	0.817	0.357	0.312
OsPIN2	Os06g44970	0.088	0.912	0.000	0.841	0.055	0.721	0.963
BKI1	Os09g28550	0.677	0.179	0.001	0.223	0.594	0.078	0.341
OsTB1/fc1	Os03g49880	0.173	0.843	0.000	0.303	0.213	0.775	0.365
GA2ox1	Os05g06670	0.344	0.264	0.000	0.922	0.368	0.264	0.921
OsAPC10	Os05g50360	0.156	0.831	0.000	0.822	0.103	0.856	0.487
D63	Os08g01110	0.111	0.908	0.000	0.568	0.260	0.281	0.203
DEP1	Os09g26999	0.247	0.159	0.000	0.774	0.113	0.370	0.641
OsFEN-1	Os05g46270	0.598	0.543	0.000	0.690	0.316	0.665	0.203
OsH1	Os03g51690	0.266	0.761	0.000	0.306	0.236	0.816	0.297

Note: Probability level equal or less than 0.05 are displayed in red. [CO_2]*N rate, [CO_2]*Organ, N rate*Organ, and [CO_2]*N rate*Organ denote the respective interaction effect of the factors.

Table S3. Significance of N metabolism related genes being altered by tissue type, CO_2 and N rate

LOC_ID#	CO_2	N rate	Organ	CO_2*Nrate	CO_2*Organ	Nrate*Organ	CO_2*N*Organ
Os09g26144	0.001	0.041	0	0.001	0.001	0.041	0.001
Os02g49340	0.001	0	0	0.003	0.001	0	0.004
Os10g40600	0.002	0	0	0.032	0.014	0.001	0.012
Os05g46840	0	0	0	0	0	0	0
Os09g26160	0	0	0.011	0	0	0	0
Os02g31080	0	0	0	0	0	0	0
Os05g48200	0	0	0	0	0	0	0
Os02g40730	0.002	0	0	0.012	0.002	0	0.021
Os04g32370	0.018	0	0	0.002	0.002	0	0.006
Os02g53130	0	0	0	0	0.001	0	0.001
Os07g37730	0	0.001	0.023	0	0.003	0	0.001
Os05g39240	0	0	0	0	0	0	0
Os04g31030	0	0	0	0	0	0	0
Os05g32820	0	0	0	0	0	0	0
Os03g63140	0	0	0	0.031	0	0	0
Os06g17870	0	0	0	0	0	0	0
Os09g25625	0.001	0.009	0	0	0.001	0.005	0
Os10g40360	0	0	0	0.002	0	0	0.003
Os08g36480	0	0	0	0	0	0	0
Os03g04370	0	0	0	0.017	0	0	0.044
Os03g48060	0	0.001	0	0	0	0	0
Os07g46460	0	0.005	0	0.001	0	0.003	0.001
Os06g40940	0.001	0	0	0	0.001	0	0
Os01g51410	0.015	0	0	0	0.011	0	0
Os04g52504	0.001	0.001	0	0.009	0.004	0.001	0.086
Os02g47850	0.004	0	0	0.037	0.015	0.011	0.646
Os03g09210	0	0.004	0	0.016	0.039	0	0.728
Os05g33510	0	0	0	0.041	0	0	0.145
Os05g26660	0	0.002	0	0.005	0	0.05	0.037
Os09g33559	0.001	0	0	0.007	0.007	0.506	0
Os06g46670	0.035	0	0	0.037	0.988	0	0.037
Os06g47000	0.01	0	0	0.002	0.139	0	0.098
Os04g40600	0.004	0	0	0.003	0.616	0.017	0.466
Os08g06430	0	0.001	0	0.002	0.109	0	0.598
Os08g04180	0	0	0	0.005	0.199	0	0.155
Os07g31460	0.003	0	0	0	0.433	0.419	0
Os01g07500	0.004	0.014	0	0.017	0.071	0.936	0.004
Os02g16630	0.011	0.001	0.002	0.02	0.328	0.175	0.636
Os08g23730	0.001	0	0	0.005	0.682	0.15	0.359
Os07g01310	0.001	0	0	0.258	0.008	0	0.034
Os07g07260	0.003	0	0	0.675	0.003	0	0.009
Os06g08900	0.01	0.002	0	0.174	0.01	0.002	0.174
Os08g36500	0.045	0	0	0.074	0.045	0	0.074
Os05g13900	0	0	0	0.471	0.001	0	0.314
Os06g09120	0.01	0	0	0.068	0.001	0	0.068
Os06g09090	0.001	0.022	0	0.481	0.031	0.027	0.131
Os01g36720	0.01	0	0	0.056	0.015	0	0.069
Os02g04170	0.019	0	0	0.669	0.015	0	0.906
Os02g22020	0.018	0	0	0.431	0.015	0.407	0.636
Os06g08890	0	0.012	0.004	0.932	0.007	0.07	0.695
Os08g04630	0.001	0.031	0	0.79	0.061	0.001	0.173
Os09g16280	0.04	0.044	0	0.369	0.061	0.017	0.524
Os01g61410	0.017	0	0	0.314	0.142	0	0.652
Os06g41390	0.037	0.012	0	0.901	0.187	0.059	0.296
Os06g09130	0.001	0.002	0	0.335	0.097	0.093	0.263
Os01g59130	0.001	0.001	0	0.066	0.322	0	0.723

Os08g15080	0	0.018	0	0.226	0.545	0.001	0.518
Os07g27790	0	0	0	0.326	0.301	0.004	0
Os12g42980	0.013	0	0	0.079	0.021	0	0
Os05g04510	0	0	0	0.146	0.001	0	0
Os05g01470	0.01	0	0	0.384	0.005	0	0.207
Os04g43070	0.014	0	0	0.504	0.013	0	0.512
Os01g22010	0	0	0	0.389	0.002	0.009	0.321
Os02g50240	0.001	0	0.085	0.957	0	0	0.005
Os03g12110	0.005	0.065	0	0	0	0	0.007
Os03g62200	0	0.057	0	0.036	0	0.031	0.044
Os01g61510	0.006	0.101	0	0.01	0.006	0.035	0.068
Os03g18130	0.002	0.83	0	0	0.003	0.004	0.162
Os03g11660	0.002	0.855	0	0.011	0	0.001	0.271
Os02g34580	0.004	0.112	0	0.006	0.005	0.122	0.008
Os10g36620	0.012	0.288	0	0.01	0.014	0.284	0.024
Os06g45670	0	0.418	0	0.006	0.001	0.976	0.023
Os03g50880	0	0.068	0.003	0.012	0	0.116	0.33
Os12g38630	0.001	0.102	0	0.01	0.917	0.001	0.581
Os02g16640	0.001	0.454	0	0.002	0.5	0.006	0.207
Os07g39710	0.002	0.898	0	0	0.177	0	0.704
Os05g43360	0	0.182	0	0.005	0.074	0.002	0.199
Os04g42095	0.008	0.065	0	0.014	0.987	0.017	0.005
Os07g48520	0.002	0.706	0	0.89	0.552	0	0.011
Os01g10950	0.014	0.35	0	0.023	0.543	0.573	0.013
Os07g25590	0.02	0.141	0	0.239	0.047	0.009	0.391
Os07g44830	0.007	0.074	0	0.148	0.006	0.039	0.19
Os03g24940	0.005	0.41	0	0.312	0.025	0.002	0.19
Os12g42876	0.022	0.244	0	0.255	0.046	0.001	0.001
Os04g40410	0.017	0.34	0	0.083	0.013	0.344	0.149
Os01g50820	0.001	0.916	0	0.982	0.049	0.118	0.133
Os06g08910	0	0.059	0	0.841	0	0.103	0.293
Os02g08270	0.002	0.418	0	0.347	0.005	0.953	0.597
Os02g33780	0.007	0.291	0	0.147	0.004	0.288	0.234
Os03g53650	0.001	0.148	0	0.515	0.002	0.421	0.261
Os10g02760	0.001	0.519	0	0.67	0.001	0.536	0.94
Os05g46830	0	0.732	0	0.858	0	0.732	0.873
Os05g40990	0.008	0.333	0	0.146	0.185	0.064	0.013
Os08g04540	0.01	0.164	0.104	0.047	0.99	0.167	0.524
Os07g12330	0.105	0.005	0	0.04	0.023	0.002	0.995
Os08g15030	0.689	0	0	0	0.043	0.001	0.748
Os10g35940	0.299	0	0	0.011	0.841	0	0.108
Os09g28110	0.432	0.02	0	0.003	0.105	0.005	0.269
Os01g59930	0.552	0	0	0.001	0.115	0	0.102
Os01g72205	0.266	0	0	0	0.231	0	0
Os03g13560	0.228	0	0	0	0.166	0	0.029
Os01g66000	0.667	0	0	0.011	0.711	0	0.014
Os06g27760	0.33	0	0	0.001	0.27	0	0.001
Os10g42840	0.951	0	0	0.018	0.517	0	0
Os10g37180	0.942	0.002	0	0.011	0.795	0.004	0.01
Os04g56400	0.077	0	0	0	0.073	0	0
Os06g15420	0.093	0.006	0	0.001	0.066	0	0.208
Os12g42884	0.94	0	0	0.041	0.22	0.02	0.051
Os07g48390	0.114	0.034	0	0.026	0.392	0.07	0.02
Os06g42560	0.218	0.002	0	0.018	0.112	0.062	0.046
Os09g23370	0.061	0	0	0	0.137	0.762	0.942
Os04g52479	0.672	0.002	0	0.049	0.691	0.812	0.742
Os02g30640	0.073	0.025	0	0.218	0.012	0.159	0.074
Os09g33510	0.434	0.001	0	0.128	0.424	0	0.006

Os05g42350	0.819	0	0.006	0.911	0.255	0	0
Os03g19890	0.069	0.021	0	0.894	0.436	0	0.011
Os06g08880	0.732	0.014	0	0.57	0.973	0.001	0.283
Os06g13730	0.306	0.044	0	0.702	0.306	0.044	0.702
Os10g06000	0.13	0.003	0	0.155	0.13	0.003	0.155
Os10g05980	0.06	0.001	0	0.499	0.061	0.001	0.433
Os02g04500	0.113	0	0	0.218	0.958	0.002	0.237
Os02g39795	0.59	0.033	0.034	0.326	0.317	0.013	0.578
Os03g29920	0.378	0.014	0	0.517	0.541	0.017	0.524
Os03g13274	0.325	0.045	0	0.493	0.449	0.001	0.497
Os06g36840	0.233	0.003	0	0.671	0.134	0	0.051
Os03g31570	0.588	0.004	0	0.904	0.771	0	0.827
Os07g33790	0.921	0	0	0.065	0.951	0.037	0.151
Os02g57990	0.919	0	0	0.26	0.08	0	0.945
Os06g05700	0.56	0.025	0	0.546	0.482	0.008	0.328
Os01g57004	0.07	0	0	0.422	0.222	0	0.358
Os01g07910	0.783	0.001	0	0.868	0.856	0.039	0.433
Os06g04650	0.17	0	0	0.061	0.953	0.001	0.988
Os10g41400	0.749	0	0	0.103	0.948	0	0.163
Os09g07920	0.081	0	0	0.264	0.346	0	0.059
Os03g24600	0.368	0	0	0.084	0.54	0.007	0.408
Os04g52950	0.143	0	0	0.451	0.855	0.661	0.054
Os11g01410	0.826	0.015	0.1	0.014	0.235	0.36	0.92
Os12g34014	0.784	0.026	0.109	0.995	0.652	0.679	0.897
Os03g48180	0.292	0	0.059	0.809	0.435	0.265	0.876
Os01g07520	0.331	0.482	0	0.001	0	0.631	0.168
Os03g12290	0.052	0.12	0	0.001	0.024	0.033	0.14
Os06g36880	0.801	0.821	0	0.002	0.992	0	0.984
Os01g10960	0.834	0.668	0	0.009	0.834	0.668	0.009
Os01g02160	0.265	0.293	0	0.021	0.279	0.278	0.02
Os07g41600	0.818	0.162	0	0.004	0.794	0.149	0.004
Os11g06150	0.207	0.277	0.013	0.044	0.51	0.261	0.688
Os01g65090	0.922	0.293	0	0.003	0.737	0.469	0.052
Os08g04560	0.315	0.168	0	0.31	0.102	0.021	0.774
Os01g65000	0.515	0.39	0	0.558	0.312	0.005	0.23
Os03g54050	0.214	0.743	0	0.134	0.569	0.019	0.151
Os05g05740	0.081	0.968	0	0.262	0.764	0.003	0.07
Os07g23640	0.071	0.538	0	0.415	0.059	0.325	0.275
Os01g18860	0.467	0.564	0.001	0.098	0.461	0.377	0.335
Os04g32800	0.578	0.171	0	0.155	0.578	0.171	0.155
Os03g50490	0.126	0.327	0	0.849	0.134	0.382	0.821
Os01g10930	0.867	0.365	0	0.928	0.697	0.779	0.987
Os05g14880	0.152	0.082	0	0.125	0.135	0.172	0.095
Os01g11010	0.96	0.063	0	0.628	0.971	0.061	0.655
Os06g01410	0.157	0.154	0	0.85	0.336	0.562	0.921
Os09g10600	0.573	0.715	0	0.152	0.763	0.696	0.181
Os02g57180	0.638	0.53	0	0.106	0.42	0.537	0.572
Os08g23810	0.822	0.595	0	0.588	0.534	0.331	0.449
Os07g45090	0.479	0.748	0	0.51	0.124	0.215	0.833
Os06g21890	0.301	0.976	0.251	0.059	0.716	0.726	0.725

Note: CO_2 level were 400 and 600 μmol mol^{-1}; N for N rate 0 and 150 Kg N ha^{-1}; Organ for leaf and shoot apical meristem (SAM).
